# Daily physical activity is negatively associated with thyroid hormone levels, inflammation, and immune system markers among men and women in the NHANES dataset

**DOI:** 10.1371/journal.pone.0270221

**Published:** 2022-07-06

**Authors:** Christopher L. Klasson, Srishti Sadhir, Herman Pontzer

**Affiliations:** 1 Trinity College, Duke University, Durham, North Carolina, United States of America; 2 Department of Evolutionary Anthropology, Duke University, Durham, North Carolina, United States of America; 3 Duke Global Health Institute, Duke University, Durham, North Carolina, United States of America; Consiglio Nazionale delle Ricerche, ITALY

## Abstract

The acute effects of exercise on metabolic energy expenditure and inflammation are well studied, but the long-term effects of regular daily physical activity on metabolic and endocrine effects are less clear. Further, prior studies investigating the impact of daily physical activity in large cohorts have generally relied on self-reported activity. Here, we used the U.S. National Health and Nutrition Examination Survey (NHANES) to investigate the relationship between daily physical activity and both thyroid and immune activity. Daily physical activity was assessed through accelerometry or accelerometry-validated survey responses. Thyroid activity was assessed from circulating levels of thyroid stimulating hormone (TSH) and thyroxine (T4). Immune function was assessed from circulating cytokines (C-reactive protein [CRP], immunoglobulin E [IgE], fibrinogen) and blood cell counts. In general linear models including body mass index, age, gender, activity and TSH as factors, active adults had a lower levels of T4 and reduced slope of the TSH:T4 relationship. Similarly, greater physical activity was associated with lower CRP and fibrinogen levels (but not IgE) and lower white blood cell, basophil, monocyte, neutrophil, and eosinophil (but not lymphocyte) counts. Daily physical activity was also associated with lower prevalence of clinically elevated CRP, WBC, and lymphocytes in a dose-response manner. These results underscore the long-term impact of daily physical activity on both systemic metabolic activity (thyroid) and on specific physiological tasks (immune). The regulatory effects of physical activity on other bodily systems are clinically relevant and should be incorporated into public health strategies promoting exercise.

## Introduction

Regular daily physical activity is among the most effective lifestyle factors for improving cardiovascular and metabolic health, fitness, mood, and cognitive function while reducing all-cause mortality [1–5]. However, the effects of physical activity on the metabolic activity of organs and tissues other than the musculoskeletal system remain unclear. Traditional, additive models of activity and expenditure assume that physical activity does not affect metabolic energy expenditure in other physiological systems [6]. However, work in recent decades has demonstrated effects of physical activity throughout the body, including immune function and inflammation [4, 7–9], stress physiology [4, 10], glucose trafficking [11, 12], and even on the central nervous system [5, 13]. Many of these studies have examined exercise or exercise interventions, or have focused on athletes [4, 14]. Those that have investigated the impact of daily physical activity in large, representative samples of adults have typically relied on self-reported activity [9], and none to our knowledge have examined thyroid hormones, which are important mediators of metabolic activity throughout the body. In this paper, we examine the association between daily physical activity and both thyroid hormone levels and immune system biomarkers in a large sample of adult men and women.

One framework for understanding the impact of physical activity on other physiological systems is the Constrained Energy Expenditure hypothesis proposed by Pontzer, which posits that increased physical activity leads to reductions in the activity of other (i.e., non-musculoskeletal) systems [15–18]. This suppression could be evident in global (i.e., whole-body) mediators of metabolic activity, such as thyroid hormone. Results from studies examining the effects of chronic exercise on resting levels of thyroid hormones have been mixed. In animal models, sustained exercise results in lower levels of T4, T3, and TSH [19–21]. Krogh et al. found sled dogs had significantly decreased levels of all three thyroid hormones after four months of vigorous physical activity [21]. In humans, Pakarinen et al. [22] reported a reduction in resting T4 levels in response to 24 weeks of intense weight lifting, but these levels returned to pre-training values when training ceased. One study of female collegiate long distance runners reported lower levels of T4, T3, and TSH than non-athletes but no differences in measurements taken before versus after competition season [23]. Female rowers in a 20-week training program showed mixed responses in TSH, T3, and T4 [24]. However, we note that human studies to date examining thyroid responses to exercise have general lasted less than 6 months. Measures of morning testosterone levels in male endurance runners suggest endocrine adjustment to physical activity may occur over longer time periods, perhaps several years [25].

Physical activity-induced suppression could also affect specific physiological systems, such as immune function [17]. Immune function, particularly inflammation and other innate immune system activity, is a compelling target for exercise-induced metabolic suppression because it is energetically costly and potentially labile [26, 27]. During exercise and immediately afterward, inflammation increases in proportion with exercise intensity [7, 28]. However, regular exercise leads to lower baseline inflammation levels and is associated with lower levels of white blood cells (WBC), neutrophils, and lymphocytes [7, 8] and a lower risk of clinical elevation in C-reactive protein (CRP) and fibrinogen (indicators of chronic inflammation), and WBC [9]. There is also evidence that, when the energetic demands of physical activity are sufficiently high, exercise-induced immune suppression can be harmful. Intense exercise workloads such as those in elite athletes increase the risk of clinically significant suppression and functional impairment collectively referred to as overtraining syndrome or relative energy deficit syndrome [7, 8, 25, 29, 30].

In this study, we examine the association between daily physical activity and both thyroid hormones and immune system activity in a large representative sample of U.S. men and women from the National Health and Nutrition Examination Survey (NHANES). Daily physical activity was measured by accelerometry or accelerometry-validated questionnaire responses. We tested the prediction that greater daily physical activity is associated with reduced immune activity and lower thyroid hormone levels. We also investigated whether daily physical activity was associated with lower prevalence of clinically elevated immune cell counts and inflammatory cytokines.

## Methods

### Dataset

Data for the study were obtained through the U.S. National Health and Nutrition Examination Survey (NHANES) conducted by the U.S. Centers for Disease Control and Prevention (CDC) [31]. The survey has conducted sampling since 1999 with data being made public in 2-year cycles. The CDC makes the recruitment protocol and study designs publicly available [31]. We examined data from 2 cycles (2003–04, 2005–06) that included accelerometry measurements of daily physical activity and from 4 cycles (1999–2006) that included validated questionnaire responses for daily physical activity, and 3 cycles (2007–2012) that included unvalidated physical activity questionnaire responses. Analyses were restricted to adults age 18–65 years, the period during which total daily energy expenditure is stable [32].

### Daily physical activity

#### Accelerometry

The ActiGraph AM-7164 was used to monitor the intensity and duration of locomotor activities in the 2003–2004 and 2005–2006 NHANES cycles by providing uniaxial measurements in one-minute periods. The accelerometers were worn at the waist for 7 days and removed only for sleeping or bathing. We analyzed accelerometry data following Wolff-Hughes et al. [33, 34], excluding subjects with fewer than 4 valid days of accelerometry recorded. A valid day was defined as having at least 10 hours of accelerometer wear time for that day. Non-wear time was defined as periods of 60 or more consecutive minutes with 0 accelerometer counts (with up to 2 minutes with less than 100 counts/min in that period).

We began by examining both total counts/day and the percentage of moderate-to-vigorous physical activity (MVPA), which was defined as greater than 2020 counts/min following Wolff-Hughes et al. [33, 34]. Both total counts and MVPA percentage were calculated using the R package *nhanesaccel* [35]. Total counts gives a measure of total physical activity workload, whereas MVPA provides a measure of activity intensity. However, we found that these measures strongly covaried (R^2^ = 0.75, p<0.0001), and visual inspection of QQ plots demonstrated that counts were more normally distributed than MVPA. Therefore, we limited our analyses to counts, which resulted in valid measures for *n* = 4979 subjects. These subjects were used to assess the association between daily physical activity and immune activity and to validate questionnaire data.

#### Physical activity questionnaires

**Fig 1:**
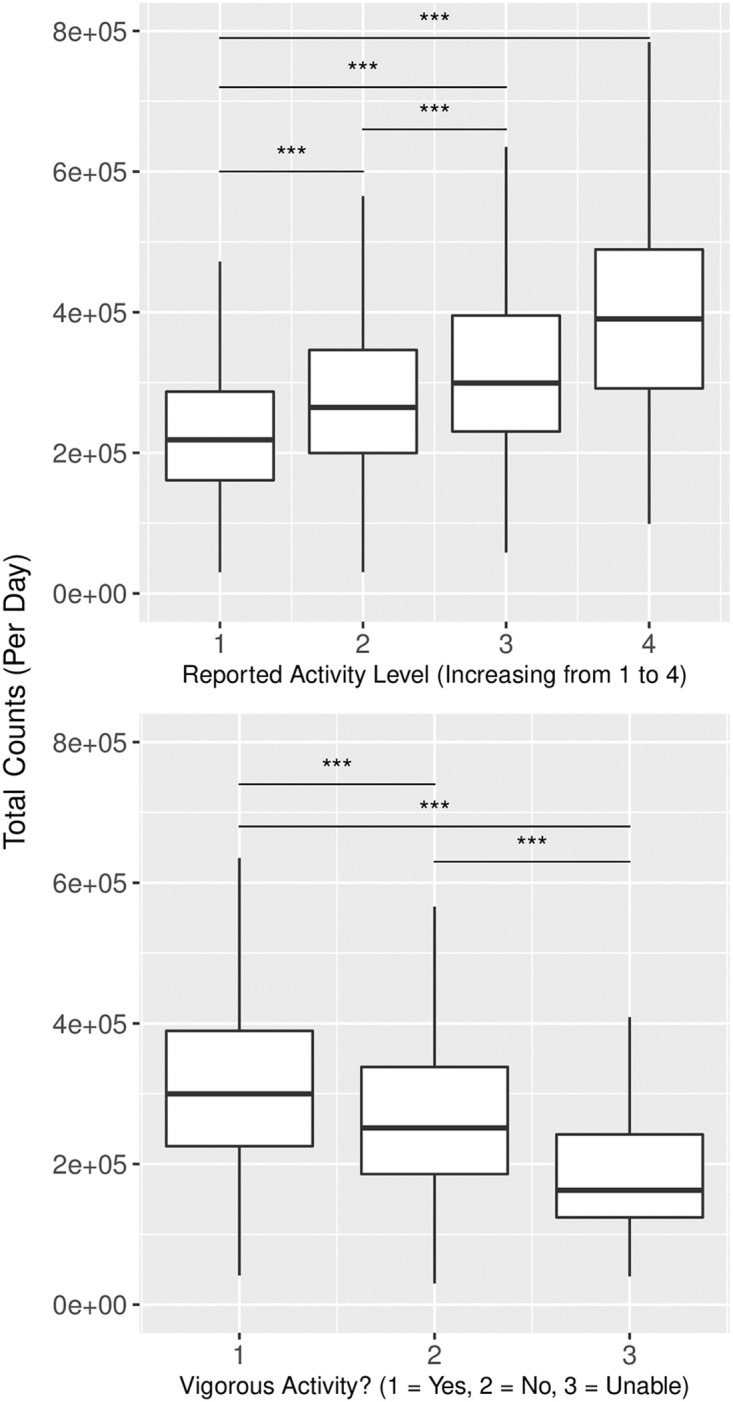
Validation of physical activity questionnaire. Physical activity questionnaire data (PAQ180 on left and PAD200 on right) displayed a significant relationship to accelerometry data indicating that self-reported answers were reflective of objectively measured activity levels. *** denotes p < 0.001.

For cycles in which there were no available accelerometer data, we estimated daily physical activity from activity questionnaires that are included in every NHANES cycle. Self-reported activity levels were validated using accelerometry data (total counts per day) in the 2003–2004 and 2005–2006 cycles using ANOVA and linear regression (n = 4979; Fig 1). Two questions proved to be significantly associated with accelerometry measures of daily physical activity. PAQ180 asks subjects to rate their overall activity level on a scale from 1 to 4, with each successive number being a higher activity level. Accelerometry measures of daily physical activity differed between each response category, in a step-wise manner (Fig 1, p < 0.001). PAD200 asked whether individuals engaged in vigorous physical activity, with answer choices of “yes,” “no,” or “unable.” Subjects answering “yes” had significantly higher accelerometry-measured physical activity than subjects answering “no” (Fig 1, p < 0.001) or those “unable” to partake physical activity (p < 0.05). For analyses of thyroid hormones and fibrinogen PAQ180, questions 3 and 4 were combined together to increase the sample size of individuals reporting high activity levels. For analysis of thyroid hormones, PAD200 was dichotomized (herein, binary PAD200) by combining the “no” and “unable” categories for engaging in vigorous physical activity. PAQ180 and PAD200 were used from the 1999–2000 through the 2005–2006 cycles (Fig 1).

### Thyroid hormones

We analyzed thyroid stimulating hormone (TSH) and thyroxine (T_4_) measurements from the 1999–2002 NHANES cycles. There are no accelerometry data for these participants, and so we examined the effect of physical activity using responses from the PAD200 and PAQ180. TSH levels were reported in IU/L and T_4_ was reported in ug/dL. We also used the 2007–2012 NHANES cycles that included responses from the PAQ650 and PAQ665, which could not be validated with accelerometry data, as a further assessment of findings from the 1999–2002 cycles.

### Immune activity

#### C-reactive protein (CRP)

Measurements of CRP, a signal of inflammation and response to infection, were available in the 2003–2004 cycles. The serum levels were measured by latex-enhanced nephelometry and reported in mg/dL [31].

#### Complete blood cell counts

White blood cell count, lymphocytes, monocytes, segmented neutrophils, basophils levels were available for the 2003–2004 and 2005–2006 cycles in the complete blood count with 5-part differential. Measurements were collected using the Beckman Coulter method of counting and sizing in conjunction with an automatic diluting and mixing device for sample processing, and a single beam photometer for hemoglobinometry. Levels were reported as a percentage and in 1000 cells/uL [31].

#### Immunoglobulin E (IgE)

IgE is generally induced in response to foreign allergens, and was measured in the 2005–2006 survey cycle. Serum levels were determined using the Pharmacia Diagnostics ImmunoCAP 1000 System and reported in kU/L [31]. While there are five immunoglobulin classes, IgE was selected for analysis as it was the only immunoglobulin class available with associated accelerometry data.

#### Fibrinogen

Fibrinogen is an important blood clotting factor that, at elevated levels, is associated with cardiovascular disease. Serum levels were determined using the Clauss clotting method in the 1999–2000 and 2001–2002 cycles for individuals aged 40 or older [31]. We analyzed fibrinogen using responses from the PAQ180 and PAD200.

### Analysis

All analyses were performed in R [Version 2021.09.01, 36]. For subjects with accelerometry data, we included mean counts per day as a continuous variable in general linear models along with age, sex, and body mass index (BMI) as covariates to test for associations with immune activity measures. We used this same approach for thyroid hormones, using binary survey response for physical activity (yes/no) as a categorical variable in general linear models along with age, sex, and BMI as covariates. All continuous variables except basophil and eosinophil counts were *ln*-transformed prior to analyses, visual inspection of QQ plots indicated that *ln-*transformed variables were more consistent with assumptions of normality. For analyses of fibrinogen, for which the survey responses for activity were not binary, we used ANCOVA with age, sex, and BMI as covariates to determine the association with activity, and used Tukey’s HSD to assess pairwise differences among questionnaire responses (R package *TukeyHSD*). Summary statistics for the accelerometry and questionnaire variables and their associated samples are provided in Tables 1 and 2.

**Table 1:** Summary statistics for the samples used for the analysis utilizing accelerometry data.

NHANES 2003–2006: Immune Measures	Men	Women
	N = 2417	N = 2562
Age (Years)	40.6 ± 14.3	40.4 ± 14.5
Body Mass (kg)	86.4 ± 20.2	75.2 ± 19.4
Body Mass Index (kg/m^2^)	28 ± 6	28.7 ± 7.08
Height (cm)	175 ± 7.82	162 ± 7.11
C-Reactive Protein (mg/dL)	0.328 ± 0.843	0.523 ± 0.789
Lymphocyte Number (1000 cell/uL)	2.14 ± 0.79	2.18 ± 7.06
Neutrophil Number (1000 cell/uL)	4.17 ± 1.66	4.53 ± 1.88
White Blood Cell Count (1000 cell/uL)	7.13 ± 2.13	7.46 ± 2.22
Monocyte Number (1000 cell/uL)	0.559 ± 0.187	0.516 ± 0.174
Basophil Number (1000 cell/uL)	0.0372 ± 0.555	0.0425 ± 0.0619
Eosinophil Number (1000 cell/uL)	0.215 ± 0.168	0.182 ± 0.144
**2005–2006: IgE Antibodies**	N = 1202	N = 1243
Age (Years)	40.7 ± 14.3	40 ± 14.2
Body Mass (kg)	87.1 ± 21.6	75.8 ± 19.4
Body Mass Index (kg/m^2^)	28.3 ± 6.5	29 ± 7.15
Height (cm)	175 ± 7.94	162 ± 6.93
IgE (kU/L)	208 ± 463	120 ± 326

Mean ± standard deviation are shown for men and women, and sample size is provided in parentheses.

**Table 2:** Summary statistics for the samples used for the analysis utilizing questionnaire responses.

NHANES 1999–2002	Men	Women
**Fibrinogen**	N = 2705	N = 2743
Age (Years)	60.1 ± 13.1	60.5 ± 13.4
Body Mass (kg)	85.1 ± 17.7	74.3 ± 18.7
Body Mass Index (kg/m^2^)	28.2 ± 5.2	29.1 ± 6.77
Height (cm)	174 ± 7.63	160 ± 7.26
Fibrinogen (mg/dL)	367 ± 82.5	385 ± 80.4
**Thyroid Hormones (1999–2002)**	N = 2072	N = 2324
Age (Years)	38.3 ± 22.5	37.7 ± 22.2
Body Mass (kg)	79 ± 20.8	69.8 ± 19.0
Body Mass Index (kg/m^2^)	26.3 ± 5.93	27.0 ± 6.75
Height (cm)	173 ± 9.01	161 ± 7.12
Free Thyroxine (ng/dL)	7.48 ± 1.61	8.39 ± 1.97
Thyroid Stimulating Hormone (IU/L)	2.13 ± 9.23	1.78 ± 2.31
**Thyroid Hormones (2007–2012)**	N = 2829	N = 2936
Age (Years)	41.4 ± 14.1	41.6 ± 13.9
Body Mass (kg)	87.5 ± 20.8	76.3 ± 21
Body Mass Index (kg/m^2^)	28.5 ± 6.19	29.2 ± 7.61
Height (cm)	175 ± 7.81	161 ± 7.15
Free Thyroxine (ng/dL)	7.62 ± 1.49	8.14 ± 1.80
Thyroid Stimulating Hormone (IU/L)	1.82 ± 1.99	2.16 ± 6.51

Mean ± standard deviation are shown for men and women, and sample size is provided in parentheses.

## Results

### Thyroid hormones

In general linear models for the 1999–2002 NHANES cycles including BMI, age, and gender as covariates, *ln*-transformed TSH was not associated with physical activity as measured by PAQ180 (p = 0.30) but did associate with physical activity as measured by three-response PAD200 (p = 0.002) and binary PAD200 (“high” versus “low” activity, p = 0.003). In similar analyses, *ln*-transformed T4 did not associate with physical activity as measured by PAQ180 (p = 0.78). However, *ln*-transformed T4 marginally trended higher for participants reporting “no” physical activity on the PAD200 (p = 0.04) and binary PAD200 (p = 0.02), but these effects did not achieve Bonferoni-adjusted significance threshold.

**Fig 2:**
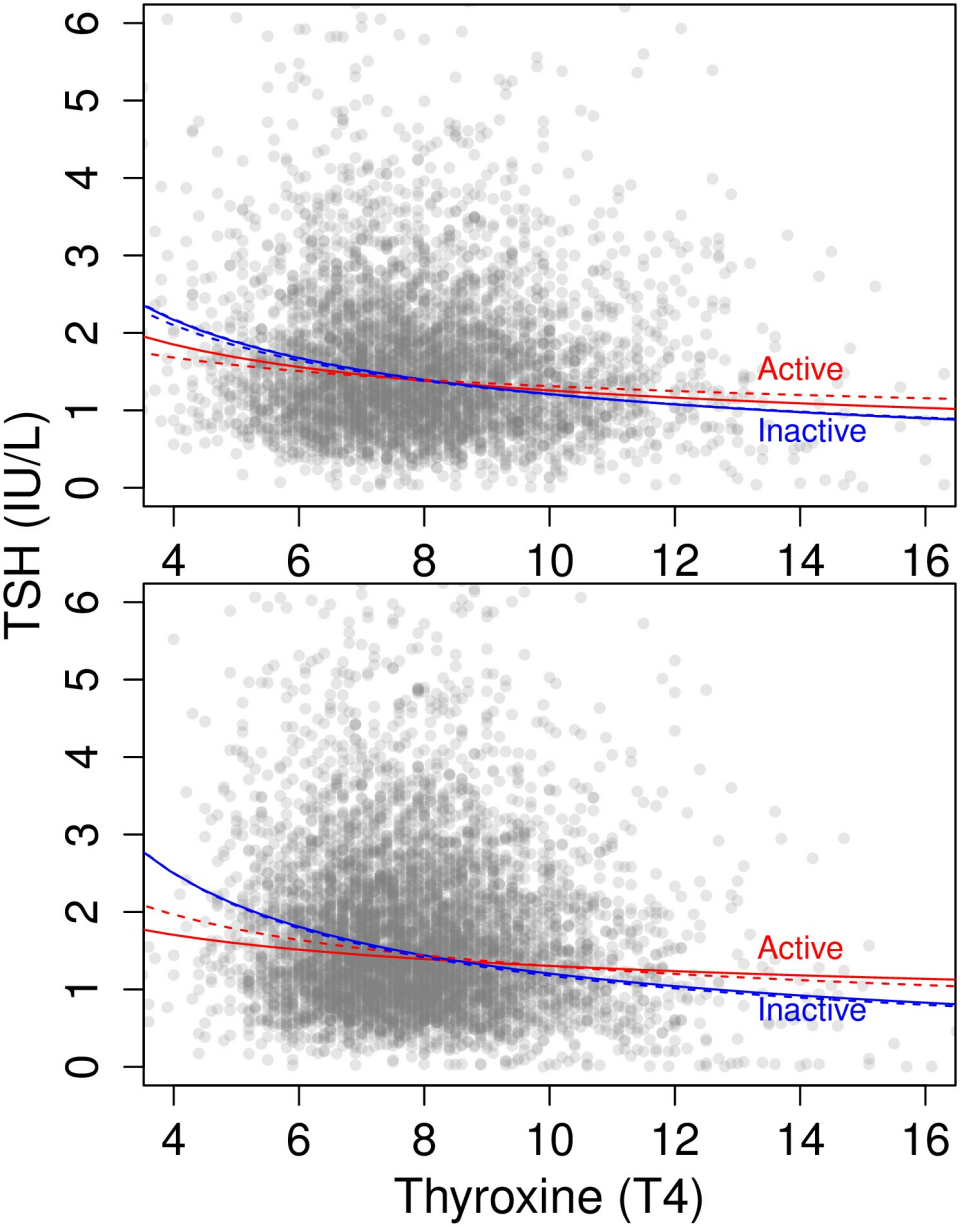
Physical activity’s relationship to thyroid hormones. The relationship between TSH and T4. The 1999–2002 data is represented by the top graph while 2007–2012 is on the bottom. Slopes for bivariate regressions of ln-transformed values shown separately for subjects reporting physical activity (“Active”, red) or no physical activity (“Inactive”, blue) in their leisure time (PAD200/PAQ650, solid line) or occupation (PAQ180/PAQ665, dashed line).

As expected, *ln*-transformed TSH and *ln*-transformed T4 were negatively correlated (p<0.0001) both in bivariate regression and in general linear models including BMI, age, and sex as covariates. Therefore, we tested whether the relationship between TSH and T4 was moderated by physical activity. In general linear models that included an interaction effect between *ln*-transformed T4 and physical activity, *ln*-transformed TSH was greater for subjects reporting “no” physical activity as measured by PAQ180 (p = 0.02), but not as measured by PAD200 (three-response: p = 0.26, binary PAD200: p = 0.10). The interaction between T4 and physical activity was significant as measured by PAQ180 (p = 0.009), but not as measured by PAD200 (p = 0.14, binary PAD200: p = 0.05). In models including an interaction effect between *ln*-transformed TSH and physical activity and T4 as the outcome variable, *ln*-transformed T4 was greater for subjects reporting “no” physical activity as measured by binary PAD200 (p = 0.02) but not as measured by PAQ180 (p = 0.83) and PAD200 (p = 0.10). The interaction between TSH and physical activity was not significant (PAQ180:p = 0.06, PAD200:p = 0.02, binary PAD200: p = 0.10). For subjects who reported “no” physical activity, the inverse relationship between T4 and TSH had a steeper slope (Fig 2).

In general linear models for the 2007–2016 NHANES cycles including BMI, age, and gender as covariates, *ln*-transformed TSH was not associated with physical activity as measured by PAQ650 (p = 0.64) or as measured by PAQ665 (p = 0.12). In similar analyses, *ln*-transformed T4 as measured by PAQ650 (p = 0.007) and PAQ665 (p = 0.01) trended higher for subjects reporting”no” physical activity, but neither met the Bonferoni-adjusted significance threshold.

Similar to the 1999–2002 analysis, *ln*-transformed TSH and *ln*-transformed T4 were negatively correlated (p<0.0001) both in bivariate regression and in general linear models including BMI, age, and sex as covariates. Thus, we again tested whether physical activity moderated the relationship between TSH and T4. In general linear models that included an interaction effect between *ln*-transformed T4 and physical activity, *ln*-transformed TSH was greater for subjects reporting “no” physical activity (PAQ650: p<0.0001, PAQ665: p = 0.0003). The interaction between T4 and physical activity was significant (PAQ650: p<0.0001, PAQ665: p = 0.0002). In models including an interaction effect between *ln*-transformed TSH and physical activity and T4 as the outcome variable, *ln*-transformed T4 was greater for subjects reporting “no” physical activity (PAQ650: p<0.0001, PAQ665: p = 0.0007). The interaction between TSH and physical activity was significant (PAQ650: p = 0.0004, PAQ665: p = 0.003).

### Immune activity

**Fig 3:**
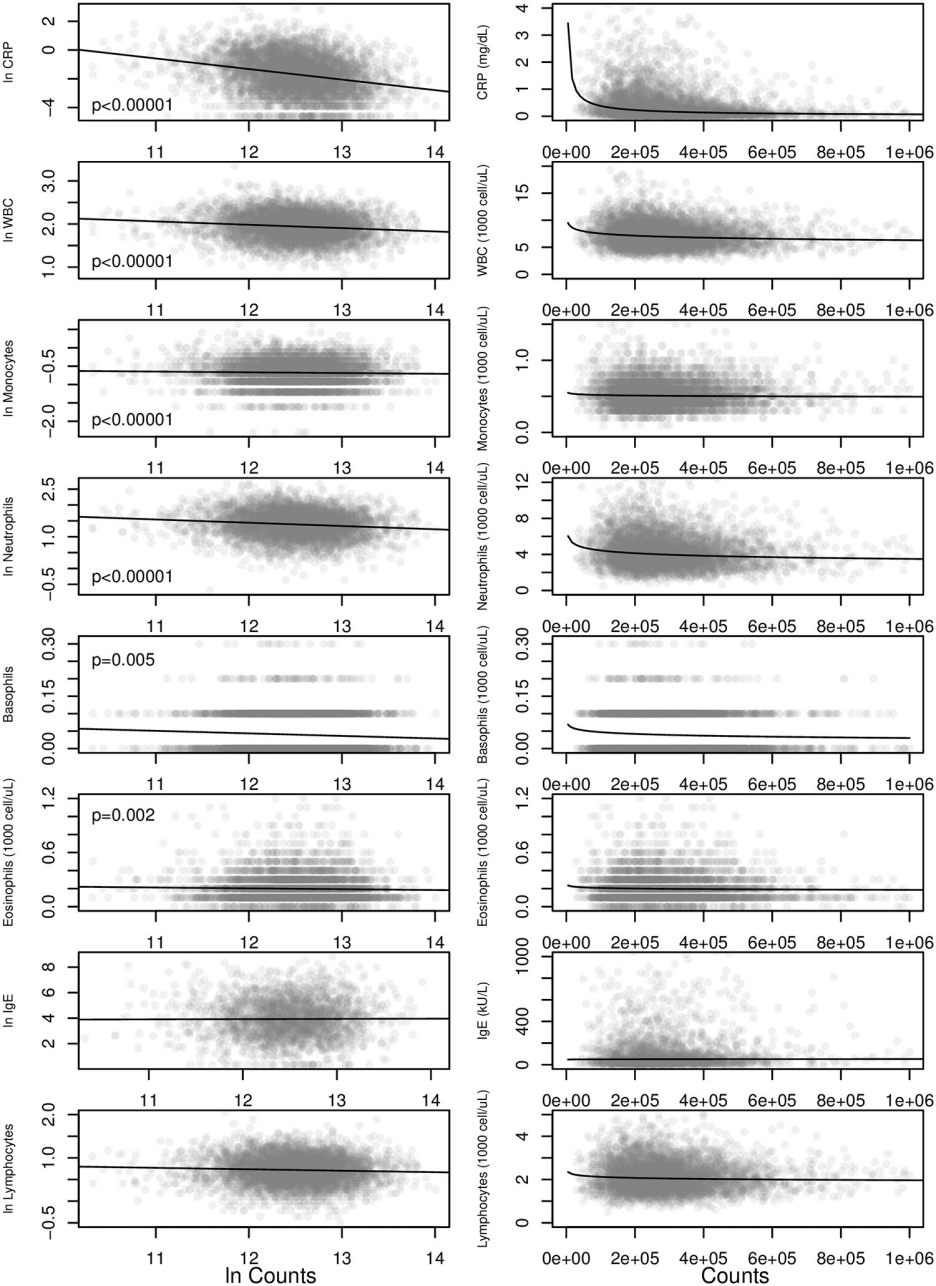
Physical activity and immune functions. Immune and inflammatory markers decrease as total counts increase. Left column shows ln-transformed marker values (except basophils and eosinophils) versus ln-transformed counts. Right column: untransformed values. P-values are for ln(counts) in general linear models with BMI, age, and gender as covariates. P-values for ln(IgE) and ln(lymphocytes) did not achieve the Bonferoni corrected significance criterion p = 0.005. Trend lines in both columns are the ordinary least squares regressions for ln-transformed values.

**Fig 4:**
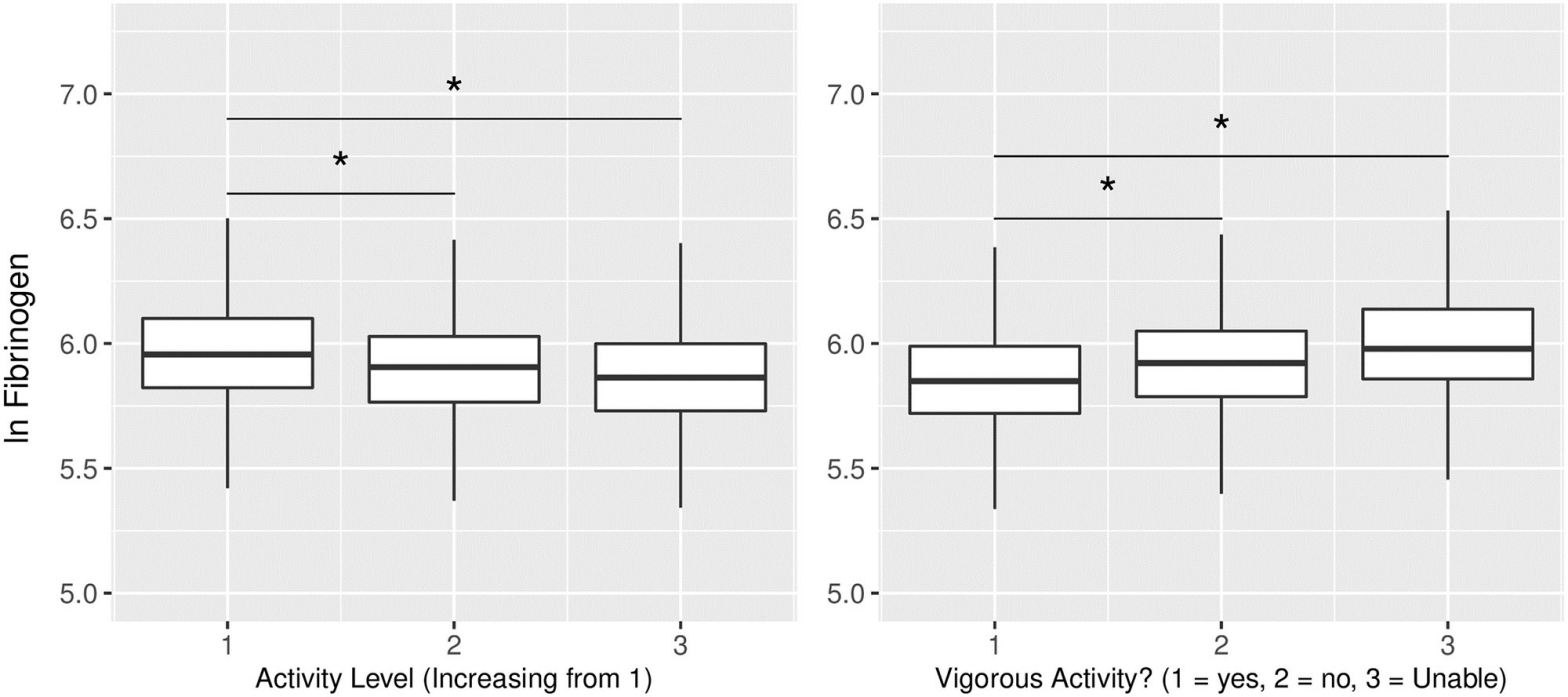
Higher physical activity relates to lower fibrinogen levels. Higher activity levels, as reported through the activity questionnaire (PAQ180 on left and PAD200 on right), were related to lower fibrinogen levels. * Denotes p < 0.05.

As predicted, measures of immune system activity were negatively associated with daily physical activity. In general linear models with BMI, sex, and age as covariates, subjects with greater total counts per day were found to have lower CRP, WBC, monocytes, neutrophils, basophils, and eosinophils (Fig 3; Table 3). Similar results were found for fibrinogen, which was lower among subjects reporting higher levels of daily physical activity in the PAQ180 and PAD200 surveys (Fig 4). The exceptions were IgE and lymphocytes, which trended lower with increased daily physical activity but did not achieve the Bonferoni-adjusted significance threshold of p = 0.005.

**Table 3:** Results from multivariable linear modelling to control for potential covariables.

Model: Predictors = BMI, Age, Years, ln(Counts)			
**Lymphocytes**	df = 4779, r-squared = 0.024, Error = ± 0.31, p < 0.0001	**CRP**	df = 4785, r-squared = 0.286, Error = ± 1.11, p < 0.0001
(Intercept)	0.894 ***	(Intercept)	-0.8565
ln(Counts)	-0.025 *	ln(Counts)	-0.337 ***
BMI	0.007 ***	BMI	0.087 ***
Age	-0.001 ***	Age	0.008 ***
Gender	0.005	Gender	0.365 ***
**White Blood Cells**	df = 4779, r-squared = 0.053, Error = ± 0.285, p < 0.0001	**Monocytes**	df = 4779, r-squared = 0.025, Error = ± 0.33, p < 0.0001
(Intercept)	2.786 ***	(Intercept)	0.08
ln(Counts)	-0.075 ***	ln(Counts)	-0.051 ***
BMI	0.006 ***	BMI	0.002 ***
Age	-0.002 ***	Age	-0.001 ***
Gender	0.019 *	Gender	-0.097 ***
**Segmented Neutrophils**	df = 4779, r-squared = 0.046, Error = ± 0.395, p < 0.0001	**Basophils**	df = 4779, r-squared = 0.004, Error = ± 0.058, p < 0.0001
(Intercept)	2.463 ***	(Intercept)	0.094 ***
ln(Counts)	-0.096 ***	ln(Counts)	-0.005 **
BMI	0.007 ***	BMI	0
Age	-0.003 ***	Age	0
Gender	0.046 ***	Gender	0.003 *
**Eosinophil**	df = 4779, r-squared = 0.016, Error = ± 0.155, p < 0.0001	**IgE Antibodies**	df = 2433, r-squared = 0.036, Error = ± 1.474, p < 0.0001
(Intercept)	0.413 ***	(Intercept)	6.073 ***
ln(Counts)	-0.016 **	ln(Counts)	-0.125
BMI	0 ***	BMI	0.016 ***
Age	0	Age	-0.005 *
Gender	-0.036 ***	Gender	-0.545 ***

Asterisks next to values for individual variables indicate significance levels:

* = p < 0.05,

** = p < 0.01,

*** = p < 0.001.

**Fig 5:**
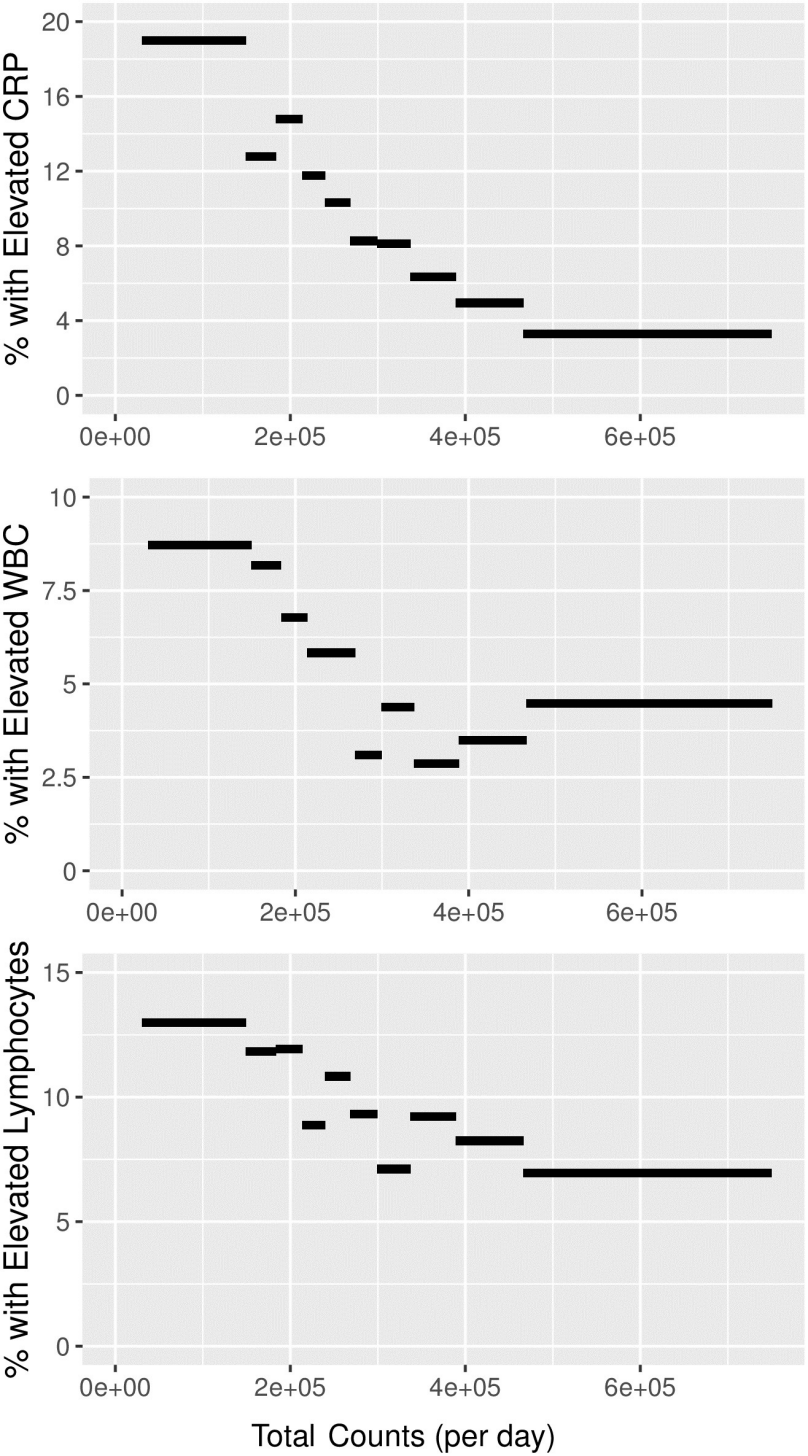
Higher physical activity corresponds to lower risk of elevated clinical serum levels. Greater daily physical activity measured using accelerometry (mean total counts per day) is associated with a lower prevalence of clinically elevated CRP, white blood cell count, and lymphocytes.

Daily physical activity was also associated with a lower prevalence of clinically elevated measures of CRP, WBC, and lymphocytes. We binned measurements by decile of physical activity and calculated the percentage of subjects with levels of these biomarkers that exceeded clinically accepted thresholds for “elevated” values. Rates of clinically elevated levels were 2–4 times higher among the lowest deciles of physical activity when compared to the highest activity deciles (Fig 5).

## Discussion

We found that U.S. adults with higher levels of daily physical activity tended to have lower levels of inflammatory cytokines (CRP and fibrinogen) and lower white blood cell counts, including lower monocytes and neutrophils (Fig 3). More active adults also tended to have lower TSH and T4 levels, and had a somewhat blunted TSH response to reduced T4 levels (Fig 2). Together, these results support the hypothesis that increasing levels of daily physical activity tends to suppress metabolic activity in other physiological systems [15–17]. We find evidence for both systemic metabolic effects via thyroid hormones and in specific systems via reduced inflammation and immune cell counts.

The association between physical activity and thyroid hormones in these cross-sectional epidemiological analyses are in line with exercise intervention studies reporting lower thyroid hormones in response to resistance and endurance training [19, 20]. We note that while the effects of physical activity on thyroid hormones were modest, explaining a small portion of the variation in TSH and T4, results were consistent across multiple NHANES cycles. Results here add to our understanding of thyroid hormone regulation by demonstrating these effects in a large, representative sample of adults under normal conditions, outside of experimental interventions. Notably, our analyses indicate that physical activity modulates not only the levels of circulating TSH and T4, but also the magnitude of TSH response to lower T4 levels. At low levels of T4, physically active adults appear to produce less TSH (Fig 2). Modulation of TSH production implicates the hypothalamic-pituitary axis, underscoring the widespread impacts of physical activity on the brain [5, 13] and suggesting that physical activity may affect metabolic energy expenditure in tissues throughout the body through effects on thyroid hormone signaling.

The negative association between physical activity with pro-inflammatory markers CRP and fibrinogen and with white blood cell counts is consistent with previous studies [7–9, 28]. The predictive power of physical activity in general linear models of inflammation and immune cell counts was small, generally <2% (Table 3). Nonetheless, the proportion of adults with clinically elevated CRP, WBC, and lymphocyte markers was clearly associated with physical activity (Fig 5). By including objective measures of physical activity from accelerometry, we are able to show that the relationship between clinical elevation and activity is essentially dose-dependent. The proportion of participants with clinically elevated levels drops with each decile of physical activity.

Contrary to our prediction, lymphocyte and IgE levels did not exhibit a statistically significant suppression. While some research suggests that chronic exercise is associated with lymphocyte suppression [8, 17, 26, 27], others find that lymphocytes are not suppressed by chronic exercise but are instead suppressed after acute exercise bouts [37, 38]. Further, previous research has not shown immunoglobulin levels to be suppressed due to exercise, but IgE specifically has not been examined prior to this study as far as we are aware [37]. Additional work, preferably in longitudinal studies rather than cross-sectional analyses, is warranted to assess the sensitivity of lymphocytes, immunoglobulin, and other immune markers to physical activity.

As with all cross-sectional analyses, this study is limited to assessing associations among variables and cannot demonstrate that physical activity is a causal factor affecting immune function, inflammation, or thyroid hormones. Additional experimental studies are needed to determine causality and ascertain the mechanisms involved. Future studies might also examine reproductive hormone levels and markers of stress reactivity, which have been shown in some previous studies to differ between sedentary and physically active adults [17]. Cortisol and testosterone levels have a strong circadian rhythm, and female reproductive hormones vary over the course of the menstrual cycle and with the use of oral contraception. Lacking the necessary context on time of day or cycle of sample collection, and for oral contraceptive use or hormone therapy, we were unable to examine those variables in this study.

This study is also limited by the use of uniaxial accelerometry (vertical acceleration) rather than triaxial accelerometry (acceleration along three planes), but there is evidence that vertical acceleration accounts for most of the variance in triaxial accelerometry counts [39]. Uniaxial accelerometry is therefore a reasonable tool to use when approximating physical activity via accelerometry, but future studies should include triaxial accelerometry to capture the full scope of movement during activity. Lastly, this analysis is framed in terms of Constrained Energy Expenditure hypothesis, but we do not have measures of metabolic rate in this analysis. Instead, we employ measurements of physical activity (accelerometry and questionnaires) as indices of energy investment in physical activity. Future work testing the relationships between physical activity, thyroid hormones, and immune function should include direct measures of metabolic rate.

Physical activity is essential for maintaining cardiometabolic and cognitive health, particularly as we age. Results from this study demonstrate the widespread regulatory effects of sustained daily physical activity in a large sample size using the NHANES data set. These findings supporting previous analyses of physical activity and inflammation while expanding our understanding of physical activity’s connections with immune and thyroid functions. We hypothesize that these changes appear due to reduced metabolic energy expenditure on other tasks resulting from exercise and may contribute to energy compensation. Regardless of their energetic mechanisms, reduced inflammation and other regulatory impacts of exercise clearly contribute to the protective benefits of exercise. Physiological models and public health strategies will continue to improve by investigating and incorporating the effects of exercise on other systems.

## Supporting information

S1 File (DOCX)
