# Supplementary material for: Daily physical activity is negatively associated with thyroid hormone levels, inflammation, and immune system markers among men and women in the NHANES dataset
Source: PLoS One. 2022 Jul 6;17(7):e0270221. doi: 10.1371/journal.pone.0270221 (PMC9258892; doi:10.1371/journal.pone.0270221)
Supplement: S1 File — (DOCX) [file pone.0270221.s001.docx]

**R Script Key for “Daily Physical Activity is Negatively Associated with**

**Thyroid Hormone Levels, Inflammation, and Immune System Markers among Men and Women in the NHANES Dataset”**

Christopher Klasson, Srishti Sadhir, Herman Pontzer

Authors for correspondence: chrisklasson98@gmail.com, herman.pontzer@duke.edu

**Code for this project can be found at OSF:**

<https://doi.org/10.17605/OSF.IO/KA73F>

**Downloading NHANES Data**

- To determine what cycle has the data you are looking for, refer to the “NHANES: 1999-2020 Survey Content Brochure” <https://wwwn.cdc.gov/nchs/data/nhanes/survey_contents.pdf>
- Go to <https://wwwn.cdc.gov/nchs/nhanes/> , and go to the “Continuous NHANES” Section and click on the NHANES cycle that has the data set you are looking for. After clicking on this, the data sets are under “Data, Documentation, Codebooks, SAS Code” under these labels: *Demographics Data*, *Dietary Data*, *Examination Data*, *Laboratory Data*, *Questionnaire Data*, and *Limited Access Data*. Once here, the XPT file can be downloaded via the link under the “Data File” tab. Further information about data sets can be found in the document under the “Doc File” column. Lastly, to upload this data into R Studio, the command “read_xpt” from the *haven* R package is used, which is included in the provided code.

**Where to find the data sets on the NHANES Website**

- All “DEMO” packages can be found under *Demographics Data*
- All “BMX” packages can be found under *Examination Data*
- All other data will be found under *Laboratory Data*
- The Letter code for many of them corresponds with the cycle they are taken from
  - No letter = 1999-2000
  - B = 2001-2002
  - C = 2003-2004
  - D = 2005-2006
  - E = 2007-2008
  - F = 2009-2010
  - G = 2011-2012

**Notable Data Sets**

- nhanes4 = primary data set for analyzing immune functions with accelerometer data
- nhanes3 = data set used to check questionnaire data with accelerometer measures
- nhanes6 = data set with ige antibodies in it

**Accelerometry Data**

- Counts = Total Counts per Day
- mvpa_percent = Moderately Vigorous Activity as Percentage of Activity

**Demographic and Body Measurement Data**

- RIDAGEYR = Age in Years
- BMXWT = Weight in Pounds
- BMXBMI = Body Mass Index
- RIAGENDR = Gender

**Questionnaire Questions**

- PAQ180
- PAD200
- .factor = addition to questions to new column made as a factor to be analyzable by TukeyHSD
- PAQ180new = column where the answer 3 and 4 were combined into 3 to increase the sample size of people who reported being highly active

**Immune Data**

- LBXCRP = C-reactive Protein (mg/dL)
- LBXWBCSI = White Blood Cells (1000 cells/uL)
- LBDLYNO = Lymphocyte Number
- LBDMONO = Monocyte Number
- LBDNENO = Segmented Neutrophils Number
- LBDEONO = Eosinophil Number
- LBDBANO = Basophil Number
- LBXIGE = IgE Antibodies
- LBXFB = Fibrinogen

**Thyroid Hormones**

- LBXT4/LBXTT4 = Thyroxine
- LBXTSH /LBXTSH1= TSH

**R Packages that you need to download**

- Nhanesaccel
  - <https://cran.r-project.org/web/packages/accelerometry/accelerometry.pdf>
- dplyr and/or dbplyr
- dvmisc
- gghalves
- ggplot2
- ggpubr
- gridExtra
- gridtext
- haven
- tidyverse
